# Role of exosomes in exacerbations of asthma and COPD: a systematic review

**DOI:** 10.3389/fmolb.2024.1356328

**Published:** 2024-06-18

**Authors:** Rossella Laitano, Luigino Calzetta, Enrico Motta, Ermanno Puxeddu, Paola Rogliani

**Affiliations:** ^1^ Unit of Respiratory Medicine, Department of Experimental Medicine, University of Rome “Tor Vergata”, Rome, Italy; ^2^ Department of Medicine and Surgery, Respiratory Disease and Lung Function Unit, University of Parma, Parma, Italy

**Keywords:** asthma, COPD, exacerbation, exosomes, systematic review

## Abstract

Asthma and chronic obstructive pulmonary disease are chronic respiratory disorders characterized by airways obstruction and chronic inflammation. Exacerbations lead to worsening of symptoms and increased airflow obstruction in both airways diseases, and they are associated with increase in local and systemic inflammation. Exosomes are cell-derived membrane vesicles containing proteins, lipids, and nucleic acids that reflect their cellular origin. Through the transfer of these molecules, exosomes act as mediators of intercellular communication. Via selective delivery of their contents to target cells, exosomes have been proved to be involved in regulation of immunity and inflammation. Although, exosomes have been extensively investigated in different diseases, little is currently known about their role in asthma and COPD pathogenesis, and particularly in exacerbations. This review aims to systemically assess the potential role of exosomes in asthma and COPD exacerbations.

## Introduction

Asthma and chronic obstructive pulmonary disease (COPD) are both chronic respiratory disorders characterized by airways obstruction and chronic inflammation (Barnes, 2008). Despite substantial differences in airways inflammation between asthma and COPD, in both conditions a wide range of inflammatory cells and mediators are involved (Barnes et al., 1998; Barnes, 2004). Among these, exosomes, extracellular nanovesicles released in the airways from immune and structural cells, were found to play a potential role in the pathogenesis of asthma and COPD inflammation (Esser et al., 2010; Kesimer et al., 2009; Almqvist et al., 2008; Tan et al., 2017). Exosomes are nanosized vesicles of 30–150 nm in diameter, found in different tissues and fluids such as blood, saliva and bronchoalveolar lavage fluid (BALF) (Yu et al., 2021; Harding et al., 1983; Admyre et al., 2003). These extracellular vesicles (EVs), enclosed by a double lipid layer, are released from different cells, including immune cells, through the fusion of multivesicular endosomes with plasma membrane (Yu et al., 2021; Torregrosa Paredes et al., 2012; Théry et al., 2009a). Exosomes contain proteins, lipids, and nucleic acids (DNAs, mRNA, miRNAs and ncRNAs) which differ depending on their cellular origin (Yu et al., 2021; Caby et al., 2005). Through the transfer of these molecules to nearby cells, exosomes act as mediators of intercellular communication, inducing a modulation of the recipient cell function (Cardoso et al., 2016a; Lässer et al., 2011; Valadi et al., 2007). Moreover, exosomes have the ability to cross all the body barriers and to transfer their cargo to remote sites (Mirershadi et al., 2020). Via selective delivery of their contents to target cells, exosomes have been proved to be involved in regulation of physiological and pathological process, such as immunity and inflammation (Théry et al., 2009a; Lee et al., 2011; Beach et al., 2014).

Although, exosomes have been extensively investigated in different diseases, such as cancer and cardiovascular diseases (Rabinowits et al., 2009; Kuwabara et al., 2011), little is currently known about their role in asthma and COPD, and particularly in exacerbations. Exacerbations are characterized by worsening of symptoms in both asthma (shortness of breath, cough, wheezing or chest tightness) and COPD (dyspnea and/or cough and sputum) along with increased airflow obstruction (GOLD, 2024; GINA, 2024). In both airways diseases, exacerbations are related with increase in local and systemic inflammation (Wenzel, 2003; Celli et al., 2021).

EVs, including exosomes, have been identified as tools of intercellular communication, involved in lung homeostasis or response to pathological developments (Lo Cicero et al., 2015a). Thus, exploring exosomes involvement in immune and inflammatory process, including exacerbations, holds great potential to understand asthma and COPD pathogenesis and to identify important biomarkers for clinical application.

Therefore, the aim of this review is to systemically assess the potential role of exosomes in asthma and COPD exacerbations.

## Materials and methods

### Review question

The question of this systematic review was to assess whether exosomes could have a potential role in asthma and COPD exacerbations.

### Search strategy

**FIGURE 1:**
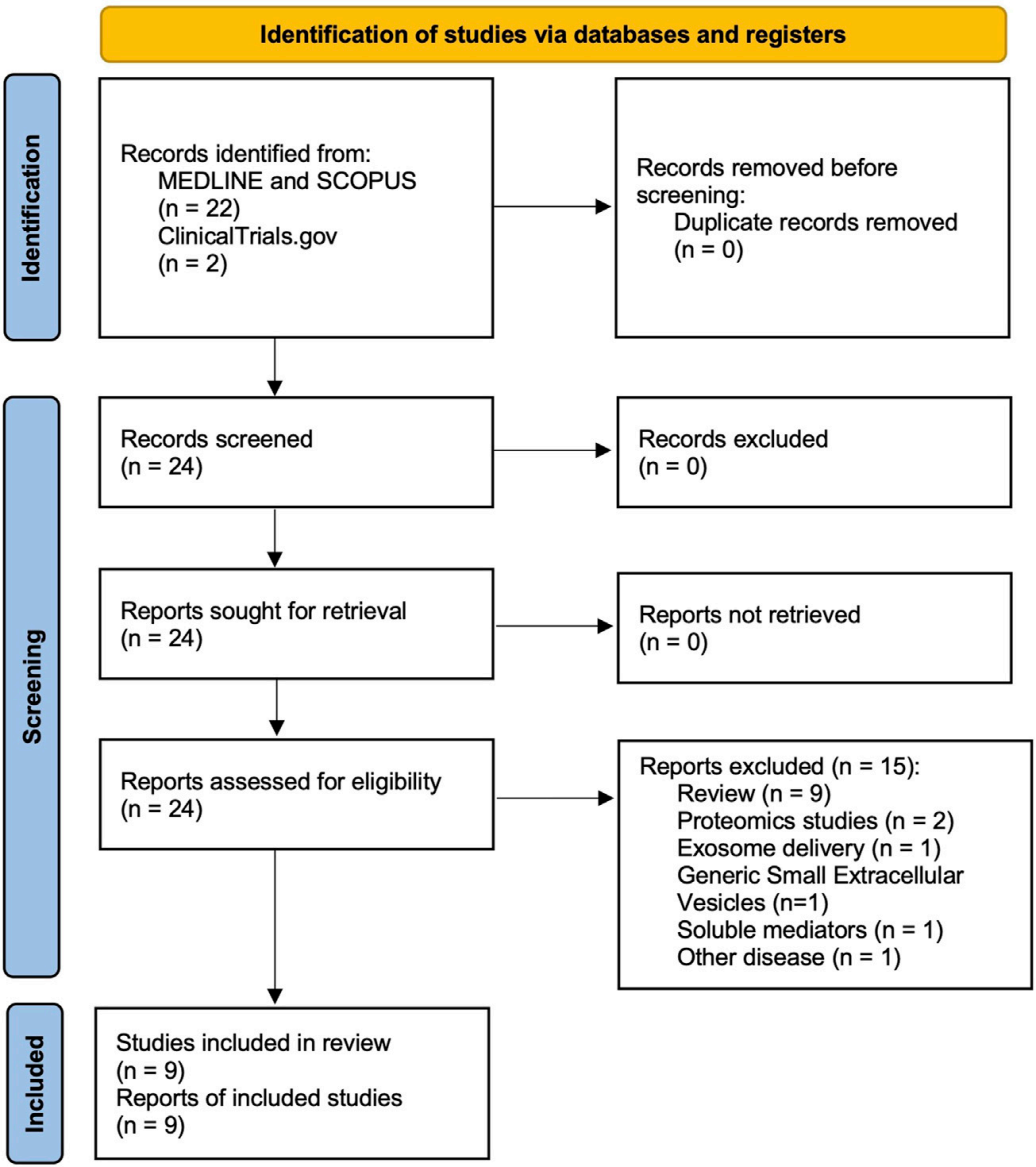
PRISMA 2020 flow diagram for the identification of the studies regarding the role of exosomes in exacerbations of asthma and COPD, resulting from databases (MEDLINE, SCOPUS) and registers (ClinicalTrials.gov) and included in the systematic review. COPD: chronic obstructive pulmonary disease. PRISMA: Preferred Reporting Items for Systematic Reviews and Meta-Analyses.

The protocol has been submitted to the international prospective register of systematic reviews (PROSPERO registration code: CRD42023483307) and performed in agreement with the Preferred Reporting Items for Systematic Reviews and Meta-Analyses Protocols (PRISMA-P) (Moher et al., 2015), with the relative flow diagram reported in Figure 1. This study satisfied all the recommended items reported by the PRISMA 2020 checklist (Page et al., 2021).

The PEO (Population, Exposure, and Outcome) framework was applied to develop the literature search strategy and question, as previously reported (Moola et al., 2015). The “Population” included asthma and COPD; the “Exposure” regarded asthma and COPD exacerbations; the assessed “Outcome” was exosomes.

A comprehensive literature search was performed for research studies, written in English, and investigating the role of exosomes in exacerbations of asthma and COPD. The search was performed in MEDLINE, Scopus, and ClinicalTrials.gov, to identify relevant studies available with no time limit up to 6 September 2023.

The string used for the search in MEDLINE, Scopus, and ClinicalTrials.gov was as follows: “exosomes AND (COPD OR asthma) AND exacerbations”.

Literature search results were uploaded to Eppi-Reviewer 4 (EPPI-Centre Software. London, United Kingdom), a web-based software program for managing and analysing data in literature reviews that facilitates collaboration among reviewers during the study selection process.

### Study selection

Research studies reporting results concerning the role of exosomes in asthma and COPD exacerbations were included in the systematic review.

Two reviewers (L.C. and R.L.) independently checked the relevant studies identified from MEDLINE, Scopus, and ClinicalTrials.gov. The studies were selected in agreement with previously mentioned criteria, and any difference in opinion about eligibility was resolved by consensus.

### Data extraction

Data from included studies were extracted and checked for study year and references, PMID or ClinicalTrials.gov identifier, study and subjects’ characteristics, outcomes, main results, and study quality assessment via the Jadad Score (Jadad et al., 1996).

### Endpoints

The endpoint of this systematic review was to evaluate the potential role of exosomes in asthma and COPD exacerbations.

### Strategy for data synthesis

Data from original papers were extracted and reported via qualitative synthesis. Statistical significance was identified for *p* < 0.05.

### Quality score

The risk of bias for included clinical studies was analyzed via the Jadad score (Jadad et al., 1996). The Jadad score, with a scale of 1–5 (score of 5 being the best quality), was used to assess the quality of the papers concerning the likelihood of bias related with randomisation, double blinding, withdrawals, and dropouts (Jadad et al., 1996). Studies were considered of low quality at Jadad score <3, of medium quality at Jadad score = 3, and of high quality at Jadad score >3.

Two reviewers (L.C. and R.L.) independently assessed the quality of studies, and any difference in opinion about the quality score was resolved by consensus.

## Results

### Study characteristics

Of the 24 potentially relevant records screened in MEDLINE, Scopus, and ClinicalTrials.gov, 9 studies were deemed eligible for a qualitative analysis. This systematic review included data obtained from studies investigating the role of exosomes in asthma and COPD exacerbations.

Seven studies (Tan et al., 2017; Yu et al., 2021; Torregrosa Paredes et al., 2012; Meng et al., 2022; Jiang et al., 2023; Song et al., 2020; Wang et al., 2022) were published in full text articles, and 2 studies (ClinicalTrials.gov, 2023b; ClinicalTrials.gov, 2023a) were available only on ClinicalTrials.gov. For 3 of the included studies (Tan et al., 2017; Torregrosa Paredes et al., 2012; Wang et al., 2022) Jadad score was suitable for quality assessment. All were considered of low quality (Jadad score = 0). The main characteristics of the studies included in the systematic review are summarized in Table 1.

**TABLE 1:** Main characteristics of the studies included in the systematic review.

Study, year and References	PMID or ClinicalTrials.gov identifier	Study characteristics	Number of analyzed subjects	Subjects characteristics	Investigated outcomes	Main results	Jadad score
Jiang et al., (2023)	37124914	Preclinical study in a mouse model of COPD	10	Male C57BL/6 mice randomly selected and divided into control and COPD groups	Exosomes, miR-7	The level of miR-7 was significantly increased in exosomes derived from COPD mice and in lung tissue macrophages COPD-exosomes induced an inflammatory response in the lungs of control mice. Lung tissue macrophages showed a shift towards M1 polarization. miR-7 inhibitor blocked M1 polarization of macrophages and reduced IL-6 and TNF-α secretion.	NA
Meng et al., (2022)	36041244	*In vivo* two-cohort study *In vitro* PM_2.5_ mice exposure model	425 (*in vivo* two-cohort study)	First cohort, 83 males and 15 females, diagnosed with stable mild-to-moderate COPD. Second cohort, 327 retirees, diagnosed with COPD and divided in ABCD group according to GOLD 2017 Male C57BL/6 mice PM_2.5_ treated group with COPD-like lesions after exposure and mice control group	Exosomal circRNA profile	PM2.5 induced and upregulated the circRNA hsa_circ_0005045 in exosomes derived from plasma, and from bronchial and alveolar epithelial cells of COPD patients In a murine COPD model, hsa_circ_0005045 homologous, through the binding to exosomal PRDX2, caused the release of TNF-α by inflammatory cells in lung tissue	NA
Wang et al., (2022)	36366542	Observational, longitudinal bi-phasic case-control study	24	Twelve subjects with mild allergic asthma and twelve well-matched non-atopic healthy controls	Circulating exosomal, MiRNAs, cytokines, chemokines, inflammatory cells in nasal lavage, FeNO, pulmonary functions parameters	At baseline, no difference in miRNA expression was observed between asthmatics and healthy controls. After the RV challenge, a total of 26 ExoMiRNAs were differentially expressed between asthmatics and healthy controls. The Upregulated Cluster miRNAs, significantly correlated with Th1 and regulatory cytokine, and Downregulated Cluster miRNAs, significantly correlated with pulmonary function measurements, inflammatory biomarkers, and Th2 and Th17 cytokine groups	0
Jin et al., 2021 (ClinicalTrials.gov, 2023b)	NCT04183530	Prospective, cohort study	NA	Patients with COPD exacerbations. Stable COPD patients. Healthy controls	Transcriptome analysis of serum or plasma, metabolomics analysis of urine or stool, proteomics analysis of BALF and saliva	NA	NA
Yu et al., (2021)	34414666	Preclinical study in a mice model of asthma	NA	Female C57BL/6J mice divided in 3 groups: control group, Asthma + PBS group, and Asthma + OAE group	OAEs	OVA challenge AECs induced the release of a major number of exosomes, compared to PBS-challenged AECs. PLXNB2, a CD100 ligand, was the most expressed protein in OAEs and in BALF exosomes from asthmatic mice compared to controls. The proteolytic cleavage of macrophages CD100, mediated by OAEs MMP14, promoted pro-inflammatory responses in the airways	0
Malakauskas et al., 2020, (ClinicalTrials.gov, 2023a)	NCT04542902	Randomized, parallel study	NA	Allergic and severe eosinophilic asthma subjects and healthy controls	ncRNA expression between eosinophils subtypes	NA	NA
Song et al., (2020)	33215438	Preclinical study in a rat model of asthma	42	Male Sprague Dawley rats. Thirty-six out of the 42 male rats divided into 6 groups	MSC-derived exosomes	MSCs and MSC-derived exosomes significantly reduced inflammatory cells in OVA-sensitized and challenged rats’ airways, and the proliferation of goblet cells and collagen deposition	NA
Tan et al., (2017)	28476471	Prospective, cohort-study	60	Patients with COPD exacerbations and stable COPD patients Ex-smokers (>15 P-Y and ceased smoking >1 year earlier) Healthy age-matched, non-smoking controls COPD treated with anticholinergics, LABA and ICS.	Circulating exosomes, CD9^+^, CRP, sTNFR1, and IL-6 plasma levels	Plasma exosomes were significantly higher in patients with COPD exacerbations and stable COPD patients compared to healthy controls Exosomes were numerically higher in patients with COPD exacerbations compared to stable COPD patients Circulating exosomes level correlated with CRP, sTNFR1, and IL-6 levels	0
Torregrosa Paredes et al., (2012)	22620679	Prospective, cohort-study	25	Healthy individuals and birch pollen–sensitized mild asthmatics subjects. Subjects with stable asthma treated with occasional medications with inhaled b2-agonist	Phenotypical and functional characteristics of BALF exosomes	Higher levels of surface molecules (tetraspanins CD81 and CD63, and HLA-DR) were observed in BALF exosomes from asthmatic patients, compared to healthy controls MUC1 decreased after allergen provocation BALF exosomes of asthmatics induced significantly higher production of LTs and IL-8, and a modest increase in IL-6 in BEC, compared to healthy controls, with no difference after allergen challenge	0

AECs, airways epithelial cells; BALF, bronchoalveolar lavage fluid; BEC, bronchial epithelial cells; COPD, chronic obstructive pulmonary disease; CRP, C-reactive protein; ELANE, exosome-bound neutrophil elastase; FeNO, fractional exhaled nitric oxide; GOLD, global initiative for chronic obstructive lung disease; ICS, inhaled corticosteroids; IL-6, Interleukin-6; IL-8, Interleukin-8; LABA, Long-Acting Beta Agonists; LTs, leukotrienes; MSC, mesenchymal stem cell; NA, not applicable; OAEs, ovalbumin-challenged airways epithelial cells-derived exosomes; P-Y, Pack-Years; PLXNB2: Plexin B2; PRDX2, peroxiredoxin2; RV, rhinovirus; sTNFR, soluble Tumor Necrosis Factor Receptor-1; TNF-α, Tumor Necrosis Factor-α.

### Role of exosomes in COPD exacerbations

In 2017, a study conducted by Tan et al. (Tan et al., 2017) described a correlation between the level of circulating exosomes and the levels of plasma biomarkers of systemic inflammation in COPD patients. Circulating exosomes, identified as CD9^+^ macrovesicles, and plasmatic systemic inflammation biomarkers, such as C-reactive protein (CRP), soluble tumor necrosis factor receptor-1 (sTNFR1) and interleukin (IL)-6, were quantified in patients with acute exacerbation of COPD (n = 20) or stable COPD (n = 20), and non-smoking healthy controls (n = 20). Plasma exosomes were significantly higher in patients with exacerbations (*p* < 0.001) and stable COPD patients (*p* < 0.05) compared to healthy controls. A numerical increase in plasma exosomes level was described in patients with exacerbations compared to stable COPD patients. Moreover, the level of circulating exosomes correlated with plasma levels of CRP (*p* < 0.001), sTNFR1 (*p* < 0.01), and IL-6 (*p* < 0.01).

Higher levels of plasma CRP and sTNFR1 were observed in patients with exacerbations compared to stable COPD patients (*p* < 0.01 and *p* < 0.05, respectively) and healthy controls (*p* < 0.001 for both correlation). In stable COPD patients, plasma CRP was higher than in healthy controls (*p* < 0.001). Also IL-6 plasma level were higher in patients with exacerbations and stable COPD patients, compared to healthy controls (*p* < 0.05 and *p* < 0.01, respectively).

Overall, the study by Tan et al. (Tan et al., 2017) suggested that circulating exosomes were elevated in both stable COPD and in patients with COPD exacerbations, and a correlation with systemic inflammatory biomarkers was demonstrated.

In 2022, Meng et al. (Meng et al., 2022) described, in an *in vitro* and *in vivo* PM_2.5_ exposure models, the effect of PM_2.5_ on exosomal hsa_circ_0005045. PM_2.5_, an ambient fine particulate matter, has been associated with higher prevalence of COPD among non-smoking subjects (Wang et al., 2018). The study showed that PM_2.5_ induced and upregulated the exosomal circRNA hsa_circ_0005045. This molecule, through the binding to the exosomal protein peroxiredoxin2 (PRDX2), induced elastase (ELANE) and tumor necrosis factor (TNF)-α release by inflammatory cells, thus exacerbating airways inflammatory response.

A cohort of 83 males and 15 females, diagnosed with stable mild-to-moderate COPD, was enrolled. A pair of blood samples, pre-exposure and post-exposure, was collected from the same subjects at 10–14 days after an air pollution episode (daily PM_2.5_ > 75 μg m^− 3^). Eight matched blood samples from this cohort were used for circRNA microarray analysis and exosomes were isolated from blood before and after PM_2.5_ exposure.

Healthy donors matched with sex, age, and smoking status were enrolled. Peripheral blood samples were collected during the stable phase in COPD patients and from healthy donors. Non-smoking was defined as smoking cessation for at least 1 year, and smokers were considered as subjects currently smoking or as having smoked at least 100 cigarettes in one’s lifetime.

Concerning *in vivo* murine model, male C57BL/6 mice treated group received PM_2.5_ for 2 weeks leading to COPD-like lesions after exposure. Mice control group received high-efficiency particulate air-filtered room air (FRA) at the same flow rate.

CircRNAs microarray analysis showed that the levels of hsa_circ_0005045 in the plasma of COPD patients were significantly elevated compared to healthy controls. From circRNAs microarray analysis, performed on plasma samples of non-smoking COPD patients who experienced exacerbation after PM_2.5_ ambient exposure, 111 upregulated circRNAs and 69 downregulated circRNAs were detected. Quantitative Reverse Transcription Polymerase Chain Reaction (qRT-PCR) was performed to confirm the upregulation of 6 specific circRNAs, including hsa_circ_0005045. Four of these circRNAs were associated with the “enrichment of extracellular exosomes” category, suggesting a correlation between circRNAs and exosomes. Analysis of the expression levels of 4 candidate exosomal circRNAs in 98 matched samples revealed that only hsa_circ_0005045 showed a significant upregulation in exosomes following exposure to PM_2.5_. hsa_circ_0005045 potentially interacted in a complex with PRDX2 and neutrophil ELANE within exosomes. Both, PRDX2 and ELANE exhibited elevated plasma concentrations following PM_2.5_ exposure, and these changes correlated with the levels of hsa_circ_0005045. Furthermore, hsa_circ_0005045 expression increased in exosomes derived from both bronchial and alveolar epithelial cells after *in vitro* exposure to PM_2.5_.

A second cohort of 327 retirees diagnosed with COPD and divided in ABCD group according to GOLD 2017 (Barnes, 2017), was recruited to predict the risk of COPD acute exacerbations, by using a machine learning model. This model showed that COPD patients sensitive to PM_2.5_ exposure were non-smoking, group C, and more likely to express higher levels of exosomal hsa_circ_0005045.

The function of hsa_circ_0005045 was further investigated *in vivo*, in a murine model with COPD-like lesions induced by PM_2.5_ inhalation. The levels of a circRNA homologous to hsa_circ_0005045 were consistently and significantly elevated after 7, 14, and 28 days of PM_2.5_ inhalation in COPD-like murine BALF and plasma-derived exosomes. Moreover, these exosomes, isolated from PM_2.5_-inhaled mice, induced hallmarks of COPD and increased plasma levels of hsa_circ_0005045 homologous in healthy murine lungs. After PM_2.5_ exposure, a significantly increase of PRDX2, inflammatory cells, and TNF-α was observed in lung tissues of both exposed and control mice, suggesting a potential contribution of the hsa_circ_0005045 homologous enriched exosomes to induce hallmarks of COPD after PM_2.5_ exposure.

Recently, Jiang et al. (Jiang et al., 2023) conducted a preclinical study, in a mouse model of COPD, with the aim to investigate the role of serum exosome-derived miR-7 in the pathogenesis of COPD. miR-7 is a MicroRNAs considered a COPD biomarker, significantly upregulated in the serum of COPD patients (Akbas et al., 2012). The study showed that the level of miR-7 was significantly (*p* < 0.0001) increased in exosomes derived from COPD mice and in lung tissue macrophages (*p* < 0.01). Furthermore, COPD-exosomes induced an inflammatory response in the lungs of control mice. Lung tissue macrophages were found to be increased and showed elevated inducible nitric oxide synthase (iNOS) and decreased Arg1 levels, indicating a shift towards M1 macrophage polarization (iNOS, *p* < 0.0001; Arg1, *p* < 0.001). Moreover, pro-inflammatory cytokine levels secreted by macrophages showed significant increases (IL-6, *p* < 0.0001; TNF-α, *p* < 0.0001). The study also tested the effect of miR-7 inhibitor that blocked M1 polarization of macrophages and reduced IL-6 and TNF-α secretion, confirming miR-7 involvement in macrophage activation and differentiation.

Overall, the study showed that elevated miR-7 levels in the serum of COPD mice could play a role in COPD exacerbation by promoting M1 polarization in lung macrophages.

A prospective cohort study, available only in ClinicalTrials.gov (NCT04183530), aimed to perform a comprehensive characterization of COPD, through multidimensional data, including exosomes evaluation (ClinicalTrials.gov, 2023b). However, no results are still available.

### Role of exosomes in asthma exacerbations

In a study by Torregrosa Paredes et al. (Torregrosa Paredes et al., 2012), phenotypical and functional characteristics of BALF exosomes were investigated in asthmatic (n = 12) and healthy (n = 13) subjects. BALF exosomes were collected from mild allergic asthmatic patients, with birch pollen specific IgE (>2 kU/l), before and 24 h after birch allergen provocation. BALF exosomes from asthmatics showed an altered phenotypic profile, compared to healthy controls, even before allergen challenge. More specifically, higher levels of the surface molecules, such as tetraspanins CD81 and CD63, and HLA-DR were observed in BALF exosomes from asthmatic patients, compared to healthy controls. The scavenger receptor CD36, known to have a function in bacterial recognition (Baranova et al., 2008), and potentially implicated in asthma exacerbations in response to bacterial infections, was higher expressed by BALF exosomes from asthmatics, compared to healthy controls. No phenotypic changes were induced by allergen provocation, except for MUC1 that decreased after challenge. Both BALF exosomes from healthy controls and asthmatics expressed leukotriene A_4_ hydrolase (LTA_4_H), leukotriene C_4_ synthase (LTC_4_S), FLAP, and 15-LO-1 that are responsible of LTA_4_ conversion to LTB_4_ and LTC_4_. Furthermore, after 48 h incubation, BALF exosomes of asthmatics induced significantly higher production of LTs and IL-8 in bronchial epithelial cells (BEC), compared to healthy controls, with no difference after allergen challenge. Only a modest increase in IL-6 production in BEC was observed, but with no significant differences between asthmatics and healthy controls.

The evidence of a pro-inflammatory altered exosome profile led to hypothesize a potential role of BALF exosomes in asthmatic inflammation.

In a preclinical mice model of asthma, Yu et al. (Yu et al., 2021) observed that ovalbumin (OVA) challenge of airways epithelial cells (AECs) induced the release of a major number of exosomes, compared to PBS-challenged AECs. OVA-challenged AEC-derived exosomes (OAEs) presented a different protein composition compared to PBS-treated AEC-derived exosomes (PAEs). More specifically, PLXNB2, a CD100 ligand, was the most expressed protein in OAEs and was found to be increased in BALF exosomes from asthmatic mice compared to controls. The proteolytic cleavage of macrophages CD100, mediated by OAEs MMP14, promoted pro-inflammatory responses in the airways, suggesting a potential mechanism of OAE-mediated asthma exacerbations. Interaction between CD100 and PLXNB2 allowed to internalize OAEs, representing a trigger to the transcription of pro-inflammatory chemokines and cytokines. Thus, OAEs were found to be able to increase airways hyper-responsiveness (AHR) and to induce the infiltration or activation of macrophages, neutrophils, and eosinophils in the airways. These observations highlighted the complex interaction between AECs and innate immune cells, powered by exosomes, in the pathogenesis of asthma.

Conversely, a preclinical study conducted by Song et al. (Song et al., 2020), evidenced the contribution of mesenchymal stem cell (MSC)-derived exosomes in the inhibition of chronic allergic inflammation, airways remodeling, and epithelial-mesenchymal transition (EMT) of airways epithelium in an asthma rat model. MSCs and MSC-derived exosomes significantly reduced inflammatory cells, such as lymphocytes, eosinophils, and neutrophils in OVA-sensitized and challenged rats’ airways. Moreover, treatment with MSCs and MSC-derived exosomes significantly reduced the proliferation of goblet cells and collagen deposition. All these effects were supposed to be mediated by exosomes highly expressed miRNAs affecting the expression of key proteins of the Wnt/β-catenin signaling pathway, implicated in EMT and airways remodeling. Indeed, treatment of rat model with BML-284, a small molecule agonist of the Wnt/β-catenin signaling pathway, reversed the downregulation of this pathway in airways epithelium, induced by MSCs and MSC-derived exosomes, exacerbating airways remodeling.

Recently, Wang et al. (Wang et al., 2022) conducted a longitudinal bi-phasic case-control study to characterize the profile of circulating exosomal microRNAs (ExoMiRNAs) in mild asthmatic patients and healthy controls, after *in vivo* rhinovirus (RV) challenge. RV is a common-cold-causing respiratory virus, notably cause of asthma exacerbations (Gern and Busse, 1999). Mild allergic asthmatic patients (n = 12) and matched non atopic healthy controls (n = 12) were recruited. Serum samples were collected before and after RV challenge. Inflammatory markers were also evaluated, including cytokines, chemokines, eosinophils, and neutrophils in nasal lavage. Furthermore, fractional exhaled nitric oxide (FeNO) and pulmonary functions parameters were measured. At baseline, no difference in miRNA expression was observed between asthmatics and healthy controls. After the RV challenge, a total of 26 ExoMiRNAs were differentially expressed (DE) between asthmatics and healthy controls. Expression of these 26 DE ExoMiRNAs not only was different before and after RV challenge in asthmatics, but it was differentiated also from miRNA expression in healthy controls, after RV challenge. Among these miRNAs, two clusters were identified. The Upregulated Cluster miRNAs, that significantly correlated with Th1 and regulatory cytokine, and Downregulated Cluster miRNAs, that significantly correlated with pulmonary function measurements, inflammatory biomarkers, and Th2 and Th17 cytokine groups. Overall, the study described the regulatory roles of ExoMiRNAs in cytokine-mediated immune response, in RV-exacerbated asthma.

A randomized study, available only in ClinicalTrials.gov (NCT04542902), was designed to perform a characterization of eosinophils derived exosomes in asthmatic and severe asthmatic patients, with the aim to investigate the role of different eosinophils subtypes in asthma pathogenesis and to further delineate asthma phenotypes (ClinicalTrials.gov, 2023a). To date, no results are still available.

## Discussion

**FIGURE 2:**
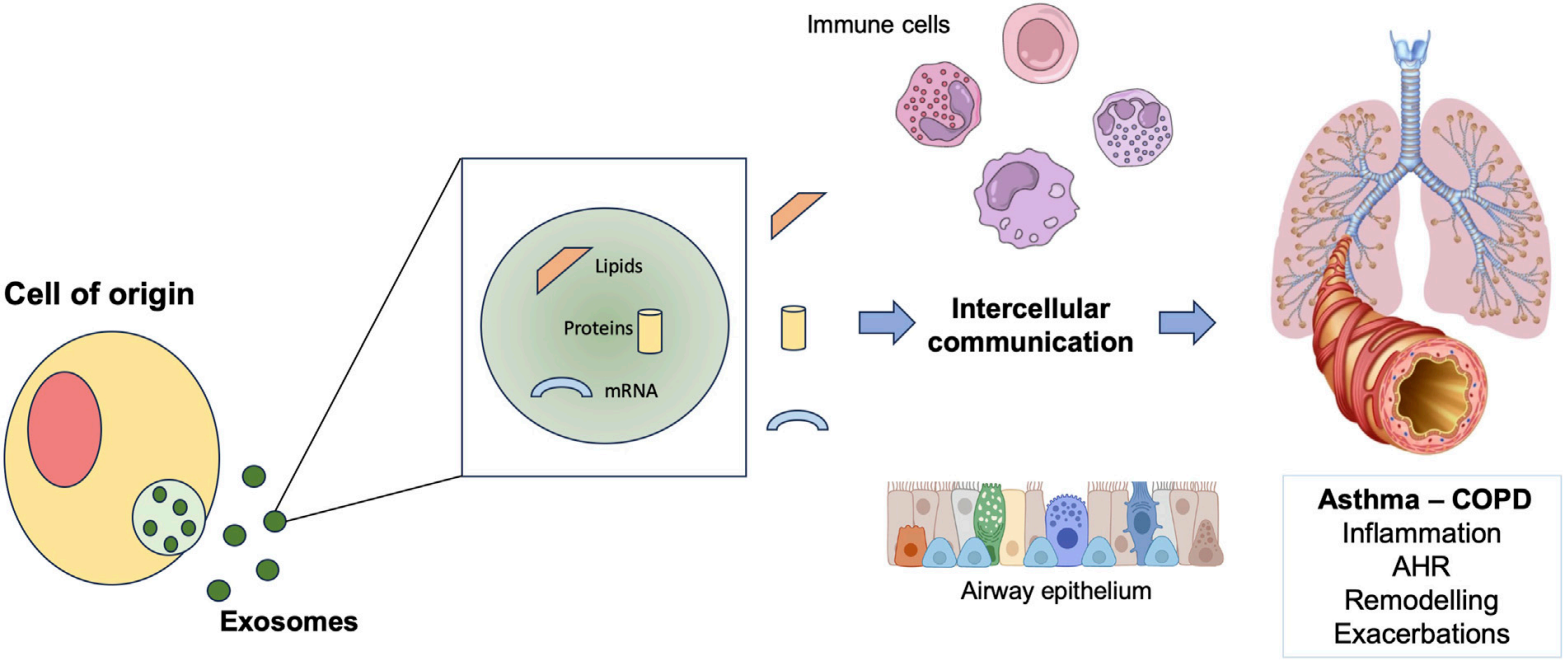
Schematic representation of the role of exosomes in exacerbations of asthma and COPD. COPD: chronic obstructive pulmonary disease; AHR: airway hyperresponsiveness.

Exosomes are extracellular nanosized vesicles released from different cell types, following the fusion of multivesicular endosomes with plasma membrane (Théry et al., 2009b). They are physiologically released but also during cellular activation, senescence, and apoptosis (Lo Cicero et al., 2015a). These cell-derived membrane vesicles are enclosed by a lipid bilayer and contain proteins, lipids, and nucleic acids. Through the transfer of these macromolecules, exosomes act as mediators of intercellular communication (Cardoso et al., 2016a). Via the activation of different signaling cascades, exosomes also play a pivotal role in inflammation process (Console et al., 2019). Exosomes are likely to be involved in modulation of inflammation also in asthma and COPD (Esser et al., 2010; Kesimer et al., 2009; Almqvist et al., 2008; Tan et al., 2017) (Figure 2). Evidence that exosomes participate to the modulation of airways inflammatory process through the release of exosomal molecules, such as nucleic acids, arise mostly from preclinical studies in asthma and COPD models. More specifically, a study conducted using both *in vitro* and *in vivo* PM_2.5_ exposure models demonstrated that PM_2.5_ induced and upregulated the circRNA hsa_circ_0005045 in exosomes derived from plasma and from bronchial and alveolar epithelial cells of COPD patients. Moreover, in a murine COPD model, the homologous of exosomal circRNA hsa_circ_0005045, binding to exosomal PRDX2, caused the release of TNF-α by inflammatory cells in lung tissue (Meng et al., 2022).

A further preclinical study, in a mouse model of COPD, proved that exosome-derived miR-7 was significantly increased in exosomes from COPD mice and was found to be involved in macrophage activation and differentiation in mice lung tissue, leading to exacerbation of local inflammatory response (Jiang et al., 2023).

Phenotypical and functional exosomes characteristics were described both in asthmatic models and patients, confirming exosomes implication in the modulation of airways inflammation in asthma, including a potential mechanism of exosomes-mediated asthma exacerbations. In a preclinical mice model of asthma, AECs released a higher number of exosomes with a peculiar protein composition, after OVA challenge. Internalization of OAEs, after CD100- PLXNB2 interaction, and proteolytic CD100 cleavage, mediated by OAEs via MMP14, triggered pro-inflammatory responses in the airways, promoting infiltration of inflammatory cells, transcription of pro-inflammatory chemokines and cytokines, and increased AHR (Yu et al., 2021). Phenotypic characteristics of asthmatics BALF exosomes were further investigated before and after allergen provocation. Asthmatics BALF exosomes showed higher levels of tetraspanins CD81 and CD63, of HLA-DR, and CD36, a scavenger receptor with a potential role in asthma exacerbations in response to bacterial infections, compared to healthy controls. Allergen provocation did not induce any exosomes phenotypic changes. Moreover, asthmatics BALF exosomes induced significantly higher production of LTs and IL-8 in BEC, compared to healthy controls (Torregrosa Paredes et al., 2012).

Conversely, in an asthma rat model, MSC-derived exosomes inhibited chronic allergic inflammation, airways remodeling, and EMT of airways epithelium. These exosomes showed high levels of miRNAs affecting the Wnt/β-catenin signaling pathway, known to be implicated in EMT and airways remodeling (Song et al., 2020).

Two clinical studies dealt specifically with the potential role of exosomes in asthma and COPD exacerbations. A clinical trial in COPD patients showed that higher levels of circulating exosomes were detectable in patients with COPD exacerbations and in stable COPD patients, compared to healthy controls. A numerical increase in plasma exosomes level was described in patients with exacerbations compared to stable COPD patients. Besides, a correlation between circulating exosomes and systemic inflammatory biomarkers was highlighted (Tan et al., 2017).

**FIGURE 3:**
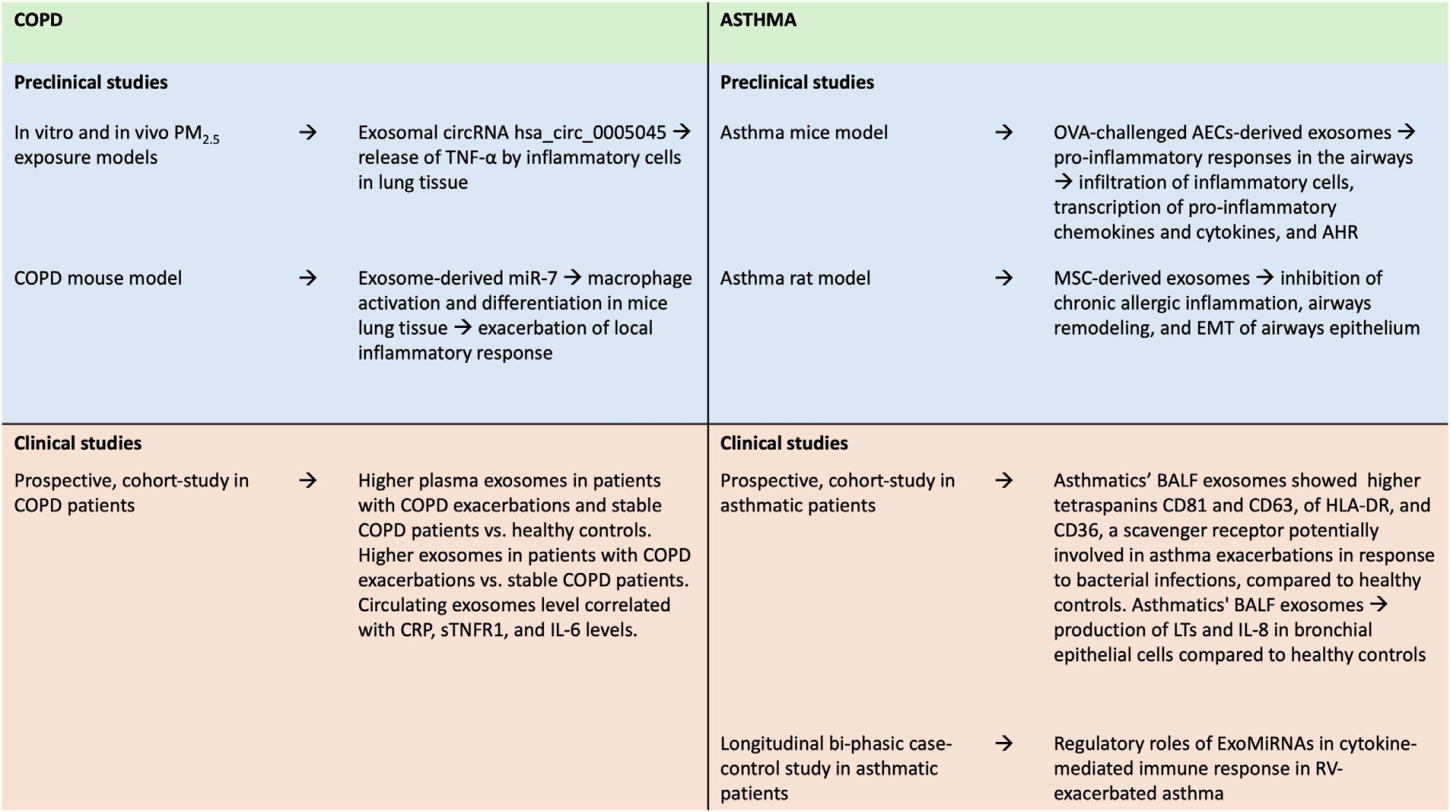
Summary of findings from preclinical and clinical studies on the role of exosomes in asthma and COPD. AECs, airways epithelial cells; AHR, airway hyperresponsiveness; BALF, bronchoalveolar lavage fluid; COPD, Chronic Obstructive Pulmonary Disease; CRP, C-reactive protein; EMT, epithelial-mesenchymal transition; IL-6, Interleukin-6; IL-8, Interleukin-8; LTs, leukotrienes; MSC: mesenchymal stem cell; OVA, ovalbumin; RV, rhinovirus; sTNFR, soluble Tumor Necrosis Factor Receptor-1; TNF-α, Tumor Necrosis Factor-α.

Recently, an observational, longitudinal bi-phasic case-control study evaluated the profile of circulating exosomal MiRNAs (ExoMiRNAs) in mild asthmatic patients and healthy controls, after *in vivo* RV challenge, providing evidence on regulatory roles of ExoMiRNAs in cytokine-mediated immune response, in RV-exacerbated asthma (Wang et al., 2022) (Figure 3).

Overall, this evidence suggests that, depending on their origin, exosomes promote inflammation via regulating the function of immune cells through their recruitment, activation, or differentiation (Engeroff and Vogel, 2022). Conversely, exosomes derived from MSCs showed regenerative proprieties in lung tissues, leading to hypothesize their potential role in attenuate remodeling in chronic lung diseases (Lener et al., 2015). Thus, cellular origin and cargo induce exosomes’ specific activity under pathological conditions (Chaput et al., 2006; Kowal and Tkach, 2019). However, current knowledge is still lacking and the process inducing pro-inflammatory or anti-inflammatory exosomes activity are under investigations (Rajabi et al., 2022).

Because of the origin from different cells type and the multitude of molecules expressed, exosomes are largely studied as an emerging class of easily accessible biomarkers (Yáñez-Mó et al., 2015; Srinivasan et al., 2016). Indeed, exosomes could provide information about their origin microenvironment, potentially useful to the diagnosis and prognosis of the diseases (Xu et al., 2016). One of the most attractive aspects of exosomes, and particularly of sputum and BALF derived exosomes, is to exploit the longitudinal sampling to monitor diseases progression (Kalluri and LeBleu, 2020). Moreover, due to expression of molecules closely linked to the pathogenesis and phenotype of specific disorders, exosomes represent a promising tool for early disease diagnosis and personalized therapy (Purghè et al., 2021).

Exosomes are characterized by considerable stability in extracellular environment, because of the lipidic bilayer, suggesting their potential application, not only as biomarkers but also as therapeutics agents (Mathis et al., 2021; Kadota et al., 2016). Engineered exosomes showed low immunogenicity and toxicity and could be able to deliver both lipophilic and hydrophilic drugs, with a preserved activity, to target cells (Raimondo et al., 2019).

Exosomes miRNA expression profile is also under investigation for diagnostic and therapeutic purposes in inflammatory respiratory disease, and targeting exosomes derived miRNA could represent a potential therapeutic strategy (Gon et al., 2020).

However, clinical application of exosomes is limited by several factors, including insufficient knowledge regarding dosage, route, timing of administration, and potential side effects. Furthermore, methods for exosomes’ isolation and purification have not been standardized, resulting in heterogeneity in exosome populations and content (Rezabakhsh et al., 2021). Currently available technologies are not able to identify exosomes secreted by specific cells within the vast number of exosomes present in body fluids. Moreover, the mechanisms of interaction between exosomes and recipient cells are not fully understood, representing a risk for potential side effects (Kadota et al., 2016). Also, the ability of exosomes to cross natural barriers (Mirershadi et al., 2020) could represent a potential risk for the uncontrolled dissemination of their contents.

An intrinsic limitation of the present systematic review is linked to limited research on the topic, therefore primary studies included in the review were heterogeneous and evidences emerged mainly from preclinical trials. Only 3 of the studies included in the present systematic review were clinical trials and were considered of low quality according to the Jadad score (Jadad score = 0).

Overall, while the biological impact of exosomes in the pathogenesis of inflammation in respiratory diseases has been widely addressed, their role in asthma and COPD exacerbations is still under investigations.

Future challenges include to investigate the cellular origin of circulating exosomes in respiratory diseases and exploring their proteomics and metabolomics content to better understand their role in acute flare-up. Randomized controlled trials, recruiting selected population, are needed to better understand the role of exosomes in exacerbations of chronic obstructive diseases.

## References

[B1] Admyre C. Grunewald J. Thyberg J. Gripenbäck S. Tornling G. Eklund A. (2003). Exosomes with major histocompatibility complex class II and co-stimulatory molecules are present in human BAL fluid. Eur. Respir. J. 22 (4), 578–583. 10.1183/09031936.03.00041703 14582906

[B2] Akbas F. Coskunpinar E. Aynaci E. Müsteri Oltulu Y. Yildiz P. (2012). Analysis of serum micro-RNAs as potential biomarker in chronic obstructive pulmonary disease. Exp. Lung Res. 38 (6), 286–294. 10.3109/01902148.2012.689088 22686440

[B3] Almqvist N. Lönnqvist A. Hultkrantz S. Rask C. Telemo E. (2008). Serum-derived exosomes from antigen-fed mice prevent allergic sensitization in a model of allergic asthma. Immunology 125 (1), 21–27. 10.1111/j.1365-2567.2008.02812.x 18355242 PMC2526256

[B4] Baranova I. N. Kurlander R. Bocharov A. V. Vishnyakova T. G. Chen Z. Remaley A. T. (2008). Role of human CD36 in bacterial recognition, phagocytosis, and pathogen-induced JNK-mediated signaling. J. Immunol. 181 (10), 7147–7156. 10.4049/JIMMUNOL.181.10.7147 18981136 PMC3842223

[B5] Barnes P. J. (2004). Mediators of chronic obstructive pulmonary disease. Pharmacol. Rev. 56 (4), 515–548. 10.1124/pr.56.4.2 15602009

[B6] Barnes P. J. (2008). Immunology of asthma and chronic obstructive pulmonary disease. Nat. Rev. Immunol. 8 (3), 183–192. 10.1038/nri2254 18274560

[B7] Barnes P. J. (2017). GOLD 2017: a new report. Chest 151 (2), 245–246. 10.1016/j.chest.2016.11.042 28183480

[B8] Barnes P. J. Fan Chung K. Page C. P. (1998). Inflammatory mediators of asthma: an update. Pharmacol. Rev. 50 (4), 515–596. 9860804

[B9] Beach A. Zhang H. G. Ratajczak M. Z. Kakar S. S. (2014). Exosomes: an overview of biogenesis, composition and role in ovarian cancer. J. Ovarian Res. 7 (1), 14. 10.1186/1757-2215-7-14 24460816 PMC3932023

[B10] Caby M. P. Lankar D. Vincendeau-Scherrer C. Raposo G. Bonnerot C. (2005). Exosomal-like vesicles are present in human blood plasma. Int. Immunol. 17 (7), 879–887. 10.1093/intimm/dxh267 15908444

[B11] Cardoso A. L. Guedes J. R. De Lima M. C. P. (2016a). Role of microRNAs in the regulation of innate immune cells under neuroinflammatory conditions. Curr. Opin. Pharmacol. 26, 1–9. 10.1016/j.coph.2015.09.001 26410391

[B13] Celli B. R. Fabbri L. M. Aaron S. D. Agusti A. Brook R. Criner G. J. (2021). An updated definition and severity classification of chronic obstructive pulmonary disease exacerbations: the rome proposal. Am. J. Respir. Crit. Care Med. 204 (11), 1251–1258. 10.1164/rccm.202108-1819PP 34570991

[B14] Chaput N. Flament C. Viaud S. Taieb J. Roux S. Spatz A. (2006). Dendritic cell derived-exosomes: biology and clinical implementations. J. Leukoc. Biol. 80 (3), 471–478. 10.1189/JLB.0206094 16809645

[B15] ClinicalTrials.gov (2023a). Study details | non-coding RNAs analysis of eosinophil subtypes in asthma. Available at: https://clinicaltrials.gov/study/NCT04542902?cond=Asthma%20OR%20COPD&term=exosome%20and&rank=2.

[B16] ClinicalTrials.gov (2023b). Study details | the individualized accurate diagnosis and treatment of chronic objective pulmonary disease(COPD) patients based on multidimensional data. Available at: https://clinicaltrials.gov/study/NCT04183530?cond=Asthma%20OR%20COPD&term=exosome%20and&rank=1.

[B17] Console L. Scalise M. Indiveri C. (2019). Exosomes in inflammation and role as biomarkers. Clin. Chim. Acta 488, 165–171. 10.1016/J.CCA.2018.11.009 30419221

[B18] Engeroff P. Vogel M. (2022). The potential of exosomes in allergy immunotherapy. Vaccines (Basel) 10 (1), 133. 10.3390/VACCINES10010133 35062793 PMC8780385

[B19] Esser J. Gehrmann U. D'Alexandri F. L. Hidalgo-Estévez A. M. Wheelock C. E. Scheynius A. (2010). Exosomes from human macrophages and dendritic cells contain enzymes for leukotriene biosynthesis and promote granulocyte migration. J. Allergy Clin. Immunol. 126 (5), 1032–1040. 10.1016/j.jaci.2010.06.039 20728205

[B20] Gern J. E. Busse W. W. (1999). Association of rhinovirus infections with asthma. Clin. Microbiol. Rev. 12 (1), 9–18. 10.1128/CMR.12.1.9 9880472 PMC88904

[B21] GINA (2024). GINA main report - global initiative for asthma - GINA. Available at: https://ginasthma.org/wp-content/uploads/2024/05/GINA-2024-Strategy-Report-24_05_22_WMS.pdf (Accessed June 06, 2024).

[B22] GOLD (2024). GOLD report - global initiative for chronic obstructive lung disease - GOLD. Available at: https://goldcopd.org/2024-gold-report/, https://goldcopd.org/wp-content/uploads/2024/02/GOLD-2024_v1.2-11Jan24_WMV.pdf (Accessed June 06, 2024).

[B23] Gon Y. Shimizu T. Mizumura K. Maruoka S. Hikichi M. (2020). Molecular techniques for respiratory diseases: MicroRNA and extracellular vesicles. Respirology 25 (2), 149–160. 10.1111/RESP.13756 31872560

[B24] Harding C. Heuser J. Stahl P. (1983). Receptor-mediated endocytosis of transferrin and recycling of the transferrin receptor in rat reticulocytes. J. Cell Biol. 97 (2), 329–339. 10.1083/jcb.97.2.329 6309857 PMC2112509

[B25] Jadad A. R. Moore R. A. Carroll D. Jenkinson C. Reynolds D. J. Gavaghan D. J. (1996). Assessing the quality of reports of randomized clinical trials: is blinding necessary? Control Clin. Trials 17 (1), 1–12. 10.1016/0197-2456(95)00134-4 8721797

[B26] Jiang Y. Wang J. Zhang H. Min Y. Gu T. (2023). Serum exosome-derived MiR-7 exacerbates chronic obstructive pulmonary disease by regulating macrophage differentiation. Iran. J. Public Health 52 (3), 563–574. 10.18502/IJPH.V52I3.12139 37124914 PMC10135496

[B27] Kadota T. Fujita Y. Yoshioka Y. Araya J. Kuwano K. Ochiya T. (2016). Extracellular vesicles in chronic obstructive pulmonary disease. Int. J. Mol. Sci. 17 (11), 1801. 10.3390/IJMS17111801 27801806 PMC5133802

[B28] Kalluri R. LeBleu V. S. (2020). The biology, function, and biomedical applications of exosomes. Science 367 (6478), eaau6977. 10.1126/SCIENCE.AAU6977 32029601 PMC7717626

[B29] Kesimer M. Scull M. Brighton B. DeMaria G. Burns K. O'Neal W. (2009). Characterization of exosome‐like vesicles released from human tracheobronchial ciliated epithelium: a possible role in innate defense. FASEB J. 23 (6), 1858–1868. 10.1096/fj.08-119131 19190083 PMC2698655

[B30] Kowal J. Tkach M. (2019). Dendritic cell extracellular vesicles. Int. Rev. Cell Mol. Biol. 349, 213–249. 10.1016/BS.IRCMB.2019.08.005 31759432

[B31] Kuwabara Y. Ono K. Horie T. Nishi H. Nagao K. Kinoshita M. (2011). Increased microRNA-1 and microRNA-133a levels in serum of patients with cardiovascular disease indicate myocardial damage. Circ. Cardiovasc Genet. 4 (4), 446–454. 10.1161/CIRCGENETICS.110.958975 21642241

[B32] Lässer C. Alikhani V. S. Ekström K. Eldh M. Paredes P. T. Bossios A. (2011). Human saliva, plasma and breast milk exosomes contain RNA: uptake by macrophages. J. Transl. Med. 9, 9. 10.1186/1479-5876-9-9 21235781 PMC3033821

[B33] Lee T. H. D’Asti E. Magnus N. Al-Nedawi K. Meehan B. Rak J. (2011). Microvesicles as mediators of intercellular communication in cancer--the emerging science of cellular debris. Seminars Immunopathol. 33 (5), 455–467. 10.1007/s00281-011-0250-3 21318413

[B34] Lener T. Gimona M. Aigner L. Börger V. Buzas E. Camussi G. (2015). Applying extracellular vesicles based therapeutics in clinical trials - an ISEV position paper. J. Extracell. Vesicles 4 (1), 30087. 10.3402/JEV.V4.30087 26725829 PMC4698466

[B35] Lo Cicero A. Stahl P. D. Raposo G. (2015a). Extracellular vesicles shuffling intercellular messages: for good or for bad. Curr. Opin. Cell Biol. 35, 69–77. 10.1016/J.CEB.2015.04.013 26001269

[B37] Mathis B. J. Kusumoto M. Zaboronok A. Hiramatsu Y. (2021). Packaging and delivery of asthma therapeutics. Pharmaceutics 14 (1), 92. 10.3390/PHARMACEUTICS14010092 35056988 PMC8777963

[B38] Meng Q. Wang J. Cui J. Li B. Wu S. Yun J. (2022). Prediction of COPD acute exacerbation in response to air pollution using exosomal circRNA profile and Machine learning. Environ. Int. 168, 107469. 10.1016/j.envint.2022.107469 36041244 PMC9939562

[B39] Mirershadi F. Ahmadi M. Rezabakhsh A. Rajabi H. Rahbarghazi R. Keyhanmanesh R. (2020). Unraveling the therapeutic effects of mesenchymal stem cells in asthma. Stem Cell Res. Ther. 11 (1), 400. 10.1186/S13287-020-01921-2 32933587 PMC7493154

[B40] Moher D. Shamseer L. Clarke M. Ghersi D. Liberati A. Petticrew M. (2015). Preferred reporting items for systematic review and meta-analysis protocols (PRISMA-P) 2015 statement. Syst. Rev. 4, 1. 10.1186/2046-4053-4-1 25554246 PMC4320440

[B41] Moola S. Munn Z. Sears K. Sfetcu R. Currie M. Lisy K. (2015). Conducting systematic reviews of association (etiology): the Joanna Briggs Institute’s approach. Int. J. Evid. Based Healthc. 13 (3), 163–169. 10.1097/XEB.0000000000000064 26262566

[B42] Page M. J. McKenzie J. E. Bossuyt P. M. Boutron I. Hoffmann T. C. Mulrow C. D. (2021). The PRISMA 2020 statement: an updated guideline for reporting systematic reviews. BMJ 372 (Mar), n71. 10.1136/BMJ.N71 33782057 PMC8005924

[B43] Purghè B. Manfredi M. Ragnoli B. Baldanzi G. Malerba M. (2021). Exosomes in chronic respiratory diseases. Biomed. Pharmacother. 144 (Dec), 112270. 10.1016/J.BIOPHA.2021.112270 34678722

[B44] Rabinowits G. Gerçel-Taylor C. Day J. M. Taylor D. D. Kloecker G. H. (2009). Exosomal microRNA: a diagnostic marker for lung cancer. Clin. Lung Cancer 10 (1), 42–46. 10.3816/CLC.2009.n.006 19289371

[B45] Raimondo S. Giavaresi G. Lorico A. Alessandro R. (2019). Extracellular vesicles as biological shuttles for targeted therapies. Int. J. Mol. Sci. 20 (8), 1848. 10.3390/IJMS20081848 30991632 PMC6514983

[B46] Rajabi H. Konyalilar N. Erkan S. Mortazavi D. Korkunc S. K. Kayalar O. (2022). Emerging role of exosomes in the pathology of chronic obstructive pulmonary diseases; destructive and therapeutic properties. Stem Cell Res. Ther. 13 (1), 144. 10.1186/S13287-022-02820-4 35379335 PMC8978512

[B47] Rezabakhsh A. Sokullu E. Rahbarghazi R. (2021). Applications, challenges and prospects of mesenchymal stem cell exosomes in regenerative medicine. Stem Cell Res. Ther. 12 (1), 521. 10.1186/S13287-021-02596-Z 34583767 PMC8478268

[B48] Song J. Zhu X. M. Wei Q. Y. (2020). MSCs reduce airway remodeling in the lungs of asthmatic rats through the Wnt/β-catenin signaling pathway. Eur. Rev. Med. Pharmacol. Sci. 24 (21), 11199–11211. 10.26355/eurrev_202011_23608 33215438

[B49] Srinivasan S. Vannberg F. O. Dixon J. B. (2016). Lymphatic transport of exosomes as a rapid route of information dissemination to the lymph node. Sci. Rep. 6, 24436. 10.1038/SREP24436 27087234 PMC4834495

[B50] Tan D. B. A. Armitage J. Teo T. H. Ong N. E. Shin H. Moodley Y. P. (2017). Elevated levels of circulating exosome in COPD patients are associated with systemic inflammation. Respir. Med. 132, 261–264. 10.1016/J.RMED.2017.04.014 28476471

[B51] Théry C. Ostrowski M. Segura E. (2009a). Membrane vesicles as conveyors of immune responses. Nat. Rev. Immunol. 9 (8), 581–593. 10.1038/nri2567 19498381

[B52] Théry C. Zitvogel L. Amigorena S. (2002). Exosomes: composition, biogenesis and function. Nat. Rev. Immunol. 2 (8), 569–579. 10.1038/nri855 12154376

[B54] Torregrosa Paredes P. Esser J. Admyre C. Nord M. Rahman Q. K. Lukic A. (2012). Bronchoalveolar lavage fluid exosomes contribute to cytokine and leukotriene production in allergic asthma. Allergy Eur. J. Allergy Clin. Immunol. 67 (7), 911–919. 10.1111/j.1398-9995.2012.02835.x 22620679

[B55] Valadi H. Ekström K. Bossios A. Sjöstrand M. Lee J. J. Lötvall J. O. (2007). Exosome-mediated transfer of mRNAs and microRNAs is a novel mechanism of genetic exchange between cells. Nat. Cell Biol. 9 (6), 654–659. 10.1038/ncb1596 17486113

[B56] Wang C. Xu J. Yang L. Xu Y. Zhang X. Bai C. (2018). Prevalence and risk factors of chronic obstructive pulmonary disease in China (the China Pulmonary Health [CPH] study): a national cross-sectional study. Lancet 391, 1706–1717. 10.1016/S0140-6736(18)30841-9 29650248

[B57] Wang W. Sinha A. Lutter R. Yang J. Ascoli C. Sterk P. J. (2022). Analysis of exosomal MicroRNA dynamics in response to rhinovirus challenge in a longitudinal case-control study of asthma. Viruses 14 (11), 2444. 10.3390/V14112444 36366542 PMC9695046

[B58] Wenzel S. (2003). Severe/fatal asthma. Chest 123, 405S-10S. 10.1378/chest.123.3_suppl.405S-a 12629003

[B59] Xu R. Greening D. W. Zhu H. J. Takahashi N. Simpson R. J. (2016). Extracellular vesicle isolation and characterization: toward clinical application. J. Clin. Invest. 126 (4), 1152–1162. 10.1172/JCI81129 27035807 PMC4811150

[B60] Yáñez-Mó M. Siljander P. R. M. Andreu Z. Zavec A. B. Borràs F. E. Buzas E. I. (2015). Biological properties of extracellular vesicles and their physiological functions. J. Extracell. Vesicles 4, 27066–27160. 10.3402/JEV.V4.27066 25979354 PMC4433489

[B61] Yu Y. Zhou Y. Di C. Zhao C. Chen J. Su W. (2021). Increased airway epithelial cell–derived exosomes activate macrophage-mediated allergic inflammation via CD100 shedding. J. Cell Mol. Med. 25 (18), 8850–8862. 10.1111/jcmm.16843 34414666 PMC8435458

